# The Relationship Between Ego Depletion and Prosocial Behavior of College Students During the COVID-19 Pandemic: The Role of Social Self-Efficacy and Personal Belief in a Just World

**DOI:** 10.3389/fpsyg.2022.801006

**Published:** 2022-04-25

**Authors:** Lu Li, Hairong Liu, Guoping Wang, Yun Chen, Long Huang

**Affiliations:** ^1^Faculty of Psychology, Beijing Normal University, Beijing, China; ^2^Beijing Key Laboratory of Applied Experimental Psychology, Faculty of Psychology, Beijing Normal University, Beijing, China; ^3^School of Humanities and Management, Wannan Medical College, Wuhu, China

**Keywords:** ego depletion, prosocial behavior, social self-efficacy, personal belief in a just world, college students, the COVID-19 pandemic

## Abstract

In the context of the COVID-19, we examined the relationship between college students’ ego depletion and their prosocial behavior. We explored the mediating role of social self-efficacy between ego depletion and prosocial behavior, we also examined the moderating role of personal belief in a just world in this relationship. 1,122 college students completed the ego depletion questionnaire, prosocial behavior questionnaire, social self-efficacy questionnaire, and personal belief in a just world questionnaire. The current findings suggested that: (1) Social self-efficacy mediated the relationship between college students’ ego depletion and their prosocial behaviors. The ego depletion of college students could be used to predict their prosocial behavior through social self-efficacy. (2) Personal belief in a just world moderated the relationship between social self-efficacy and prosocial behavior.

## Introduction

Ego depletion is a process in which individual self-control resources are consumed in large quantities. After a period of activities that require self-control resources, self-control ability will be exhausted, and this state is ego depletion (Hagger et al., 2010; Chen et al., 2011; Ding et al., 2020a). The individual’s cognition, emotion, and behavioral issues are the aftereffects of ego depletion (Chen et al., 2011), such as the reduction of prosocial behavior (Fennis, 2011; Osgood and Muraven, 2015; Yi et al., 2021), the increase in online flaming, online cheating behavior and aggressive behavior, and future anxiety (Ding et al., 2020a,b; AlHarbi et al., 2021; Wang et al., 2021a).

The core idea behind ego depletion is that the self’s acts of volition draw on some limited resource, and the effects of ego depletion are maladaptive and detrimental to performance (Baumeister et al., 1998, 2007; Muraven et al., 1998). The strength model of self-control suggests that engaging in initial self-control tasks depletes self-control resources, at least partially, leading to fewer resources being available to perform subsequent tasks (Hagger et al., 2010).

Self-control is related to behaviors that meet social expectations and the pursuit of personal achievement. Based on the strength model of self-control, individual’s self-control resources are limited, when the individual is in a state of ego depletion, the depletion effect will occur (Wang et al., 2021b). Especially, according to the strength model, self-control is a finite resource that determines capacity for effortful control over dominant responses. What is worse, once the limited resource is expended, the individual’s performance in self-control tasks will be seriously weakened (Hagger et al., 2010). Prosocial behavior is often a behavior under the superego standard, which requires people to overcome selfishness and the pursuit of self-interest, and do things that are morally encouraged. This behavior requires individuals to use cognitive control to maintain. The ego depletion will lead to individuals not having enough cognitive control ability to control and restrain their behavior, which will lead to the decline of the individual’s preference for prosocial behavior.

Many studies have proved the relation between ego depletion and prosocial behavior. For example, Osgood and Muraven (2015) found that ego depletion can damage individual’s prosocial behavior by reducing their ability or motivation to overcome self-desires. Furthermore, Fei et al. (2016) recruited 58 college students and adopted the dual-task paradigm of ego depletion to investigate the effect of ego depletion on the prosocial behaviors. The results showed that college students with a high degree of ego depletion showed fewer prosocial behaviors than college students with a low degree of ego depletion. Fennis (2011) also found that ego depletion can reduce college students’ prosocial behavior of perspective taking (as a specific form of prosocial behavior). Ren et al. (2014) used the stroop task to generate ego depletion to manipulate the level of self-control of college students and observed whether the prosocial behaviors in the dictator game was affected by ego depletion. The experimental results showed that compared with the subjects in the non-ego depletion group, those in the ego depletion group showed fewer prosocial behaviors.

In addition, the negative emotions caused by the COVID-19 pandemic would further increase the level of people’s ego depletion (Caldas et al., 2021; Robert and Vandenberghe, 2021). Robert and Vandenberghe conducted a study of 650 civil servants in the Quebec government during the first wave of the COVID-19 pandemic. They examined the effect of anxiety caused by the COVID-19 pandemic on ego depletion, which found that the anxiety caused by the COVID-19 pandemic was positively correlated with ego depletion. It could be seen that individuals are more prone to ego depletion during the COVID-19 pandemic, and more ego depletion may lead to fewer prosocial behaviors. Nevertheless, society needs people to show more prosocial behaviors to jointly resist the COVID-19 during the pandemic. Therefore, it is particularly important to investigate the impact of ego depletion on prosocial behavior during the pandemic of COVID-19.

When individuals have cognitive loss, they are more likely to question their psychological capital, such as self-efficacy, and will subjectively think that they do not have enough efficacy to act. There are some studies promoting the association (Chow et al., 2015), while others find no significant association (Hagger et al., 2010). Chow et al. (2015) used three experiments to suggest that ego depletion undermines self-control by reducing the motivation (an important protective factor of self-control) to mobilize cognitive resources. One potential cognitive mechanism is the reduction of self-efficacy. Specifically, ego depletion could demotivate self-control by making people believe that they are inefficacious in exerting self-control in subsequent tasks (Chow et al., 2015). In other words, ego depletion can reduce the individual’s self-efficacy. However, Hagger et al. (2010) did not find a relationship between self-efficacy and ego depletion. This may be because self-efficacy was the independent variable and ego depletion was the dependent variable in their study. In Chow et al. (2015)‘s study, ego depletion was the independent variable and self-efficacy was the dependent variable. It can be speculated that ego depletion will lead to the decline of self-efficacy, but the level of self-efficacy cannot affect ego depletion. Social self-efficacy is a manifestation of self-efficacy in the social field, which is a special internal resource. Social self-efficacy affects the establishment and maintenance of individual interpersonal relationships in social situations, as well as the application and exertion of their interpersonal abilities (Gu et al., 2014; Zheng et al., 2019). Based on this, we hypothesized that ego depletion would be linked with social self-efficacy.

The theory of social cognition proposes that the human self-system can play a role in controlling and regulating behavior and can affect people’s choice of behavioral activities and social environment, as well as cognitive and behavioral methods (Gong et al., 2021). Individuals with high self-efficacy will recognize themselves more, compare their existing knowledge and experience with the current situation, and believe that they have enough ability to solve the problems in a positive way. Underestimate the damage or believe that the benefits outweigh the disadvantages, making individuals more inclined to perform prosocial behaviors (Gong et al., 2021). The positive connection between self-efficacy and prosocial behavior has been confirmed and verified in numerous studies. Deng et al. (2018) have conducted a questionnaire survey on 768 junior high school students from Grade one to Grade three in Shandong province and Chongqing province, their results indicated that self-efficacy was the most predictive of prosocial behavior. Gong et al. (2021) have selected 320 college students for investigation and research and found that the higher the self-efficacy of college students, the easier it is for them to implement prosocial behaviors. Patrick et al. (2018) concluded that social self-efficacy could be associated with certain types of prosocial behavior in a survey of a sample of 338 adolescents, which is considered to provide confidence for adolescents to participate in prosocial behavior. Furthermore, as mentioned above, studies have found that self-efficacy plays a mediating role in the impact of ego depletion on self-control (Chow et al., 2015). Based on above analysis, we speculated that social self-efficacy was positively related to individual’s prosocial behavior. In summary, social self-efficacy may play a mediating role between ego depletion and prosocial behavior.

Individuals need to believe in a just world (BJW) in which everybody gets what they deserve, because this enables them to deal with their physical and social environment, as if it were stable and orderly (Lerner, 1980; Dalbert and Stoeber, 2006). Individuals with high level of BJW are better able to cope with the anger-evoking situations, and BJW can be seen as a personal resource to protect not only mental but also physical health (Dalbert, 2002). Personal belief in a just world is an important dimension of belief in a just world, which means that individuals believe that the world is fair to them and that they can be treated fairly (Lipkus et al., 1996; Zhou and Guo, 2013; Liu et al., 2020a). General belief in a just world refers to people’s belief that the world is just in a general sense (Dalbert, 1999; Tian et al., 2019). It has been shown that individuals tend to endorse personal BJW more strongly than general BJW (Dalbert, 1999; Dalbert and Stoeber, 2006). Personal BJW is positively correlated with willingness to help others. Individuals who hold a high personal BJW have more confidence in the future and are more inclined to use fair means to achieve their goals (Ji et al., 2014). Whereas, general BJW is positively correlated with severe social attitudes and social discrimination, and negatively correlated with helping behavior (Bègue et al., 2008; Ji et al., 2014). Therefore, current study focus on the role of personal BJW in prosocial behavior.

**Figure 1:**
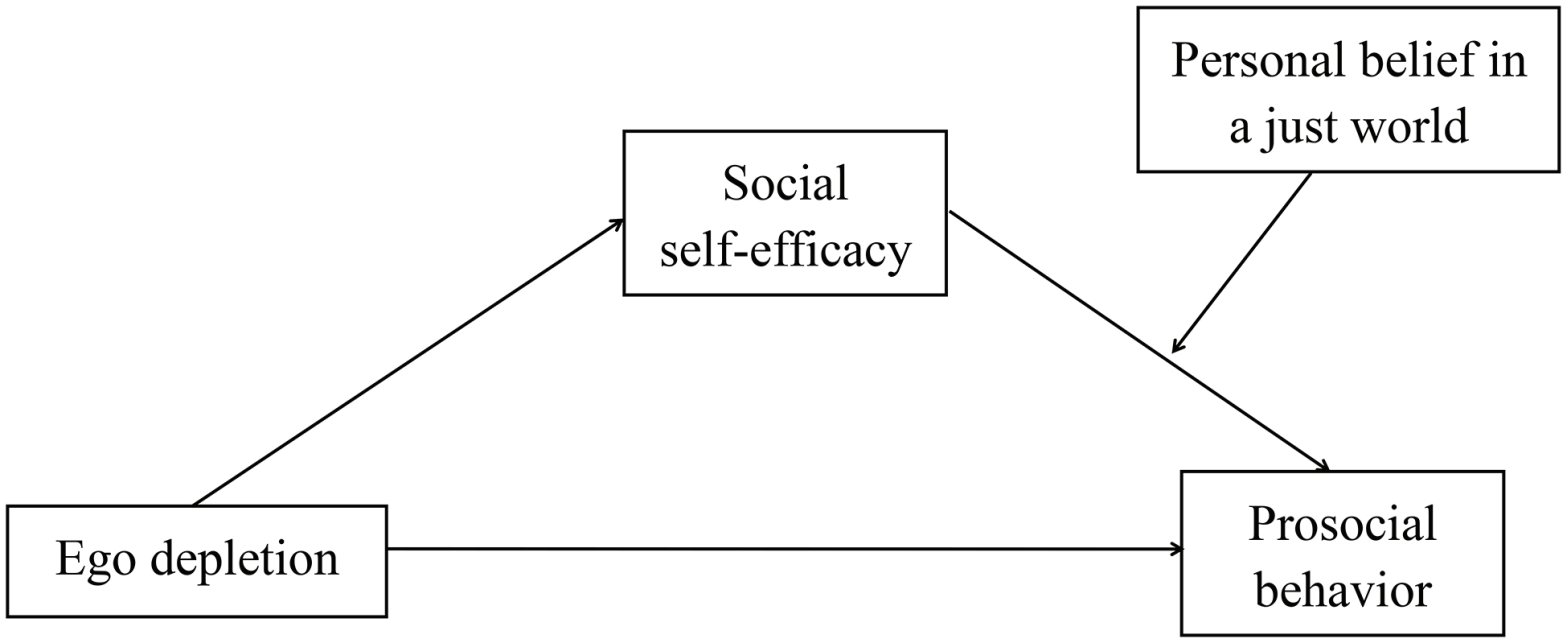
The proposed moderated mediation model.

If an individual has strong personal BJW, he will firmly believe that his current contributions will not be rewarded immediately, but he will be rewarded in the future. So when someone asks for help, he is more likely to act prosocial (Cook and Rice, 1987). From the perspective of fairness, only by helping others can you get help from others when you need it. This could suggest that the stronger personal BJW, the stronger his willingness to help others. In other words, individuals who hold strong personal beliefs in a just world are more likely to perform prosocial behaviors. Quan (2020) analyzed a sample of 960 adults who completed the prosocial behavior questionnaire and the personal just world belief questionnaire and found that personal BJW is more related to prosocial behavior. The higher personal BJW, the greater tendency of individuals to implement prosocial behaviors. Moreover, social self-efficacy can enhance the confidence of individuals to implement prosocial behaviors (Patrick et al., 2018). That is to say, personal BJW could enhance the positive connection between social self-efficacy and prosocial behavior (Figure 1).

## Materials and Methods

### Procedure and Participants

An online survey was conducted among 1,189 college students. We deleted data for participants who completed the questionnaires within 3 min and who did not complete the questionnaires. And, 1,122 college students (including 493 full-time college students and 629 part-time college students) completed these questionnaires in Central China during the COVID-19 pandemic period (April–May 2021). Participants were informed that they could terminate their participation in the online questionnaire at any time. Moreover, participants were also informed that the online survey followed the principles of anonymity, confidentiality, and independence, and the data will only be used for academic survey.

There were 293 male college students and 829 female college students; 407 first-year university students, 216 s-year university students, 206 third-year university students, and 293 fourth-year university students. The average age of the participants was 24.11 ± 5.96 years old.

### Measurement

#### Ego Depletion Scale

We used the ego depletion scale compiled by Nes et al. (2013) and revised by Wang et al. (2015) to measure the ego depletion level. This scale includes 16 items with rating on a 5-point Likert scale from 1 (strongly disagree) to 5 (strongly agree). Higher scores indicated greater levels of ego depletion. A sample item is: “I find it difficult to exercise as much as I should.” This ego depletion scale was widely used among college students in China (Chen and Xiao, 2020; Ho et al., 2020; Wang et al., 2020; Wang et al., 2021b). In current study, the Cronbach’s α of this scale was 0.79.

#### Prosocial Behavior Scale

We used the prosocial behavior scale compiled by Carlo and Randall (2002) and revised by Kou et al. (2007) to measure the prosocial behavior tendency of college students. This scale includes 26 items with rating on a 5-point Likert scale from 1 (totally inconsistent) to 5 (totally consistent). Higher score indicated stronger levels of prosocial behavior. A sample item is: “I think that helping others without them knowing is the best type of situation.” This prosocial behavior scale was often used among college students in China (Liu et al., 2020b; Zhang et al., 2020b; Fang and Chang, 2021; Gao et al., 2021; Lv and Zhou, 2021). In current study, Cronbach’s α of this scale was 0.93.

#### Perceived Social Self-Efficacy Scale

The social self-efficacy scale was first compiled by Smith and Betz (2000), which revised by Meng et al. (2007) in China. We used the revised version of social self-efficacy to measure participants’ social self-efficacy. This scale includes 18 items with rating on a 5-point Likert scale from 1 (no confidence at all) to 5 (very confidence). Higher scores indicated greater levels of social self-efficacy. A sample item is: “Ask a group of people who you do not know and who are engaging in a social activity if you can join them.” This perceived social self-efficacy scale was widely used among college students in China (Meng et al., 2007, 2012; Liu et al., 2020c; Zhang et al., 2020a). In current study, Cronbach’s α of this scale was 0.96.

#### Personal Belief in a Just World Scale

Participants’ personal beliefs in a just world were measured by using the personal BJW scale compiled by Dalbert and Stoeber (2006)and revised by Su et al. (2012). This scale includes 7 items with rating on a 6-point Likert scale from 1 (totally disagree) to 6 (totally agree). Higher scores indicated stronger levels of personal BJW. A sample item is: “I am usually treated fairly.” This personal beliefs in a just world scale was widely used among college students in China (Tian et al., 2017; Li et al., 2019; Chen, 2021; Wang et al., 2021c). In current study, Cronbach’s α of this scale was 0.86.

## Results

### Control and Inspection of Common Method Deviation

The data collected in this survey mainly came from the self-reports of participants, which may cause common method deviations. We adopted procedural control methods to minimize the impact of common method deviations, such as anonymous questionnaire surveys and balanced item order. In addition, in order to further examine whether common method bias exists, we used the Harman single factor method to carry out the common method deviation test. There were a total of 11 factors whose characteristic roots were greater than 1, and the explanatory rate of the first factor was 16.47% (<40%), which indicated that the common method bias was not serious (Podsakoff et al., 2012).

### Preliminary Analyses

We have reported the mean, standard deviation, and correlation coefficients of ego depletion, social self-efficacy, personal BJW, and prosocial behavior in Table 1. From the data summarized in the table, it could be seen that the participants’ ego depletion was significantly negatively correlated with their social self-efficacy, personal BJW, and prosocial behavior. Participants’ social self-efficacy was significantly positively correlated with personal BJW and prosocial behavior. Moreover, the participants’ personal BJW was significantly positively correlated with prosocial behavior. Post-hoc Power analysis found that statistical test power was greater than 0.99.

**Table 1:** Descriptive statistics and correlation analysis of the variables involved in this measurement (*N* = 1,122).

Variables	*M*	*SD*	1	2	3	4
Ego depletion	2.76	0.48	–			
Social self-efficacy	3.24	0.65	−0.25^***^	–		
personal belief in a just world	4.24	0.74	−0.34^***^	0.31^***^	–	
prosocial behavior	3.65	0.45	−0.20^***^	0.35^***^	0.42^***^	–

*** *p* < 0.001.

### The Relationship Between College Students’ Ego Depletion and Their Prosocial Behavior: A Test of Mediation Effect

We used the PROCESS 3.4 (Hayes, 2013) to analyze the mediation model. Ego depletion was used as the independent variable, social self-efficacy was used as the mediating variable, and prosocial behavior was used as the dependent variable. We used Model 4 and Bootstrap method (sample size is 5,000, 95% confidence interval) to test the significance of the mediation effect (see Table 2).

**Table 2:** The mediating effect of social self-efficacy between ego depletion and prosocial behavior.

Predictors	Equation 1 (variable: Social self-efficacy)			Equation 2 (variable: Prosocial behavior)			Equation 3 (variable: Prosocial behavior)		
	*β*	*t*	95% CI	*β*	*t*	95% CI	*β*	*t*	95% CI
Ego depletion	−0.25	−8.58^***^	[−0.41, −0.26]	−0.20	−6.77^***^	[−0.24, −0.13]	−0.12	−4.16^**^	[−0.31, −0.18]
Social self-efficacy							0.32	11.00^***^	[0.25, 0.38]
*R* ^2^	0.06			0.04			0.13		
*F*	73.67^***^			45.79^***^			85.89^***^		

Standardized values were used for all variables.

*** p < 0.001

** p < 0.01.

Ego depletion is significantly negatively associated with social self-efficacy (*β* = −0.25, *t* = −8.58, *p* < 0.001), and social self-efficacy is significantly positively linked to prosocial behavior (*β* = 0.32, *t* = 11.00, *p* < 0.001). Furthermore, ego depletion is still significantly negatively connected with the participants’ prosocial behavior (*β* = −0.12, *t* = −4.16, *p* < 0.001). Post-hoc Power analysis found that statistical test power was greater than 0.99. This result suggested that social self-efficacy played a mediating role between ego depletion and prosocial behavior of the participants. For further verification, we also draw 5,000 samples to estimate the 95% confidence interval of the mediation effect. The indirect effect of ego depletion on prosocial behavior, that is, the Bootstrap 95% confidence interval of ego depletion on social self-efficacy was [−0.31, −0.18] (alpha = 0.05), the Bootstrap 95% confidence interval of social self-efficacy for prosocial behavior was [0.25, 0.38] (alpha = 0.05). In summary, the interval did not contain a value of 0, indicating that social self-efficacy had a significant mediating effect in ego depletion and prosocial behavior among participants. Moreover, the Bootstrap 95% confidence interval of the direct effect of ego depletion on prosocial behavior is [−0.24, −0.13] (alpha = 0.05), and the interval did not contain a value of 0. This shows that participants’ social self-efficacy could play a mediating role between ego depletion and prosocial behavior. And, total effect of ego depletion on prosocial behavior is −0.20 (*t* = −6.77, *p* < 0.001), direct effect of ego depletion on prosocial behavior is −0.12 (*t* = −4.16, *p* < 0.001).

### Test of the Moderating Effect of Personal BJW Between Social Self-Efficacy and Prosocial Behavior

We use PROCESS 3.4 (Hayes, 2013) to further analyze the moderating effect of personal BJW. Ego depletion was used as the independent variable, social self-efficacy was used as the mediator, prosocial behavior was used as the dependent variable, and personal BJW was used as the moderator. And we running Model 14 and Bootstrap method (sample size is 5,000, 95% confidence interval) to test the moderating effect of personal BJW. The interaction of personal BJW and social self-efficacy was significantly related to college students’ prosocial behavior (*β* = 0.08, *t* = 3.13, *p* < 0.01, bootstrap 95% confidence interval was [0.02, 0.13], alpha = 0.05, see Table 3), indicating that personal BJW moderated the relationship between social self-efficacy and prosocial behavior. *R*^2^ = 0.23, *F* = 84.51, *p* < 0.001. Post-hoc Power analysis found that statistical test power was greater than 0.99. So, personal BJW had a significant moderating effect between social self-efficacy and prosocial behavior. And direct effect of ego depletion on prosocial behavior is −0.03 (*t* = − 1.13, *p =* 0.26), index of moderated mediation is −0.02([−0.03, −0.00]; 95% confidence interval, alpha = 0.05).

**Table 3:** The moderating effect of personal BJW between social self-efficacy and prosocial behavior.

Predictors	Equation 1 (variable: Social self-efficacy)			Equation 2 (variable: Prosocial behavior)			Equation 3 (variable: Prosocial behavior)		
	*β*	*t*	95% CI	*β*	*t*	95% CI	*β*	*t*	95% CI
Ego depletion	−0.25	−8.58^***^	[−0.41, −0.26]	−0.20	−6.77^***^	[−0.24, −0.13]	−0.03	−1.13	[−0.09, 0.24]
Social self-efficacy							0.23	8.19^***^	[0.17, 0.28]
Personal BJW							0.33	11.26^***^	[0.27, 0.38]
Social self-efficacy × Personal BJW							0.08	3.13^**^	[0.03, 0.12]
*R* ^2^	0.06			0.04			0.23		
*F*	73.67^***^			45.79^***^			84.51^***^		

Standardized values were used for all variables.

*** p < 0.001

** p < 0.01.

**Figure 2:**
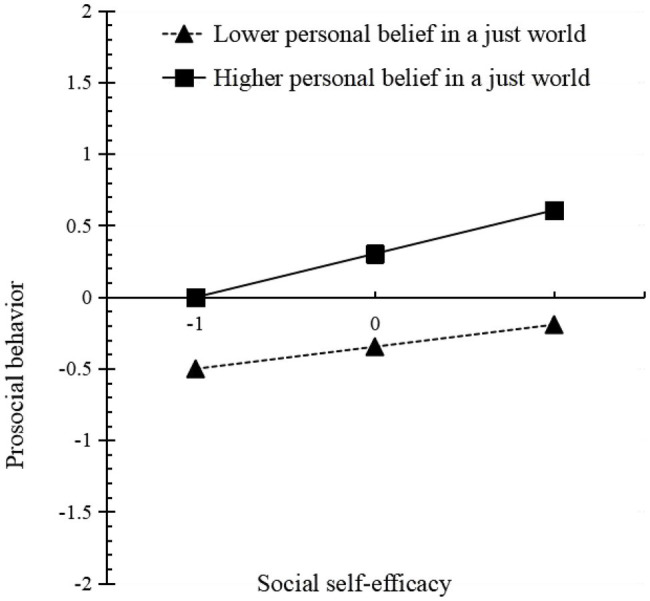
The moderation of personal BJW between social self-efficacy and prosocial behavior.

Participants were divided into a lower group (*Z* = −1) and a higher group (*Z* = 1) based on the standard scores of personal BJW. A simple slope test was used to examine the impact of social self-efficacy on prosocial behaviors of participants who hold different levels of personal BJW. Participants who hold a lower level of personal BJW, social self-efficacy could predict prosocial behavior significantly (*β*_simple_ = 0.15, *t* = 4.00, *p* < 0.001, Bootstrap 95% confidence interval was [0.08, 0.23], alpha = 0.05); Participants who hold a higher level of personal BJW, their social self-efficacy would be connected with prosocial behavior more significant (*β*_simple_ = 0.30, *t* = 8.68, *p* < 0.001, Bootstrap 95% confidence interval was [0.24, 0.37], alpha = 0.05). On the whole, the mediating role of social self-efficacy in ego depletion and prosocial behavior was moderated by the level of participants’ BJW (Figure 2).

## Discussion

The current study revealed the relationship between ego depletion and prosocial behavior of college students during the COVID-19 pandemic, as well as the mediating role of social self-efficacy and the moderating role of personal BJW through a moderated mediation model.

The current study found that social self-efficacy mediates the relationship between ego depletion and prosocial behavior, which is consistent with the Strength Model of Self-control. Based on the strength model of self-control, self-control resources are limited. When individual is in a state of self-depletion, individuals would have ego depletion effect (Wang et al., 2021b). Our study also found a negative correlation between self-depletion and self-efficacy. The more self-depletion, the lower the self-efficacy, which is consistent with previous research results (Chow et al., 2015). Moreover, based on the theory of social cognition (human self-system could play a role in controlling and regulating behavior, and can influence its choice of behavioral activities and behavior; Gong et al., 2021), individuals with high self-efficacy would consider themselves competent enough to solve the problems encountered in a positive way, and thus are more inclined to perform prosocial behaviors (Gong et al., 2021). Our investigation of the positive relationship between social self-efficacy and prosocial behavior during the COVID-19 pandemic was more consistent with previous research conclusions (Deng et al., 2018; Patrick et al., 2018; Gong et al., 2021). For example, research conducted by Patrick et al. (2018)‘s study suggested that social self-efficacy could be associated with certain types of prosocial behaviors. Thus we can see that social self-efficacy plays an indispensable role in enhancing prosocial behaviors. Therefore, improving the establishment and maintenance of individual interpersonal relationships in social situations and the application and exertion of interpersonal skills can increase the possibility of prosocial behaviors. In short, college students’ ego depletion can be associated with their prosocial behaviors through their social self-efficacy during the COVID-19 pandemic.

The moderating effect of personal BJW between social self-efficacy and prosocial behavior was also verified. Current research suggested that when individuals hold strong personal BJW, the positive connection between social self-efficacy and prosocial behavior was stronger. At a motivational level, personal BJW is related to social goals that require the suspension of immediate self-interest. Personal BJW shows associations with the human motivational values of benevolence, as well as a desire to learn more about others, talk about feelings, and make others feel better (Bartholomaeus and Strelan, 2019). If someone asks for help, individuals are more likely to be willing to perform prosocial behavior, when they have the confidence to participate in prosocial actions (Patrick et al., 2018). We may also be able to understand the moderating effect of personal BJW between social self-efficacy and prosocial behavior from the perspective of the resource conservation model. The model of resource conservation stated that people strive to retain, protect, and build resources, and that what is threatening to them is the potential or actual loss of these valued resources (Hobfoll, 1989). That is to say, individuals already possess some resources and will often strive to acquire, maintain, and protect resources that they consider valuable (Hu and Shen, 2020). Individuals with more resources are less susceptible to resource loss attacks and are more capable of obtaining resources, thus forming a value-added spiral (Hobfoll et al., 2003; Hu and Shen, 2020). Prosocial behavior is often an act of helping others at the expense of one’s own interests. Therefore, from the perspective of personal material gain, prosocial behavior is often accompanied by losses (Schwartz, 2010; Patrick et al., 2018). Personal BJW can be understood as additional resource making this losses more bearable. In conclusion, personal BJW can enhance the positive connection between social self-efficacy and prosocial behavior. This result suggests that we should pay attention to the difference in the strength of personal BJW when intervening to improve individuals’ prosocial behavior tendencies by enhancing individual social self-efficacy.

Our investigation has enriched the application of the strength model of self-control and the theory of social cognition in the relationship between ego depletion and prosocial behavior during the COVID-19 pandemic, which has positive enlightening significance for how to enhance people’ s prosocial behaviors during the COVID-19 pandemic.

We can start by reducing the ego depletion of college students. Based on the ego depletion theory, when individuals modify the way they think, feel, or behave to adapt to societal norms and expectations, they draw from a limited pool of regulatory resources (Baumeister et al., 2000; Caldas et al., 2021). If individuals draw from this pool too much, this results in resource depletion that ultimately “renders the self temporarily less able and less willing to function normally or optimally” (Baumeister et al., 2007; Caldas et al., 2021). We need to take concrete action to mitigate the ego depletion among college students during the COVID-19 pandemic. For instance, we can encourage college students to actively participate in repeated practice on self-control tasks (e.g., regulating mood and monitoring eating habits), which have been proved to be effective in alleviating ego depletion (Hagger et al., 2010).

## Limitations

In addition, this survey could still be improved in the following aspects: Firstly, Since this survey was a cross-sectional study, it could not make causal inferences about the relationship among college students’ ego depletion, prosocial behaviors, social self-efficacy, and personal BJW. Secondly, what we reveal was the relationship between ego depletion and prosocial behaviors of college students during the COVID-19 pandemic. This kind of prosocial behavior is holistic, and the relationship between ego depletion and specific dimensions of prosocial behavior (such as anonymity) would be investigated in the future, which will provide us with more detailed information about the relationship between ego depletion and prosocial behavior. Thirdly, we have discussed the mediating role of social self-efficacy, a sense of self-efficacy in the social field, between ego depletion and prosocial behavior. The possible role of other special self-efficacy between ego depletion and prosocial behavior still has value to study. Finally, the discussion on the relationship between ego depletion and prosocial behaviors during the COVID-19 pandemic was mainly based on college students. The research objects could be further expanded to increase the reliability of the conclusions.

## Conclusion

College students’ ego depletion could reduce their prosocial behaviors, which has been confirmed by many studies during regular periods (Fennis, 2011; Ren et al., 2014; Osgood and Muraven, 2015; Fei et al., 2016). The current study further enriches this result with an online questionnaire survey on the relationship between ego depletion and prosocial behaviors of college students during the COVID-19 pandemic. The current study found that the ego depletion of college students could be linked to their prosocial behaviors through social self-efficacy. Moreover, the stronger the personal BJW held by college students, the more significant the connection between their social self-efficacy and prosocial behaviors.

## Data Availability Statement

The raw data supporting the conclusions of this article will be made available by the authors, without undue reservation.

## Ethics Statement

The studies involving human participants were reviewed and approved by Academic Committee of Wannan Medical College. The patients/participants provided their written informed consent to participate in this study.

## Author Contributions

LH and LL designed the survey. HL, CY and GW collected data. LL wrote the manuscript. LH and CY revised the manuscript. All authors contributed to the article and approved the submitted version.

## Funding

This study was supported by the Philosophy and Social Science Planning Project of Anhui Provincial (Grants No. AHSKQ2019D059).

## Conflict of Interest

The authors declare that the research was conducted in the absence of any commercial or financial relationships that could be construed as a potential conflict of interest.

## Publisher’s Note

All claims expressed in this article are solely those of the authors and do not necessarily represent those of their affiliated organizations, or those of the publisher, the editors and the reviewers. Any product that may be evaluated in this article, or claim that may be made by its manufacturer, is not guaranteed or endorsed by the publisher.
